# Subchronic toxicity, immunoregulation and anti-breast tumor effect of Nordamnacantal, an anthraquinone extracted from the stems of *Morinda citrifolia* L

**DOI:** 10.1186/s12906-018-2102-3

**Published:** 2018-01-27

**Authors:** Nadiah Abu, Nur Rizi Zamberi, Swee Keong Yeap, Noraini Nordin, Nurul Elyani Mohamad, Muhammad Firdaus Romli, Nurulfazlina Edayah Rasol, Tamilselvan Subramani, Nor Hadiani Ismail, Noorjahan Banu Alitheen

**Affiliations:** 1 UKM Molecular Biology Institute (UMBI), UKM Medical Center, Jalan Yaacob Latif, Bandar Tun Razak 56000 Cheras, Kuala Lumpur, Malaysia; 2 Department of Cell and Molecular Biology, Faculty of Biotechnology and Biomolecular Science, Universiti Putra Malaysia, 43400 Serdang, Malaysia; 3 China-ASEAN College of Marine Sciences, Xiamen University Malaysia, 43900 Sepang, Selangor Malaysia; 4 Atta-ur-Rahman Institute for Natural Products Discovery, Universiti Teknologi MARA, 40450 Puncak Alam, Selangor Malaysia

**Keywords:** Nordamnacanthal, *Morinda citrifolia*, Breast cancer, 4T1, Immunomodulation

## Abstract

**Background:**

*Morinda citrifolia* L. that was reported with immunomodulating and cytotoxic effects has been traditionally used to treat multiple illnesses including cancer. An anthraquinone derived from fruits of *Morinda citrifolia* L., nordamnacanthal, is a promising agent possessing several in vitro biological activities. However, the in vivo anti-tumor effects and the safety profile of nordamnacanthal are yet to be evaluated.

**Methods:**

In vitro cytotoxicity of nordamnacanthal was tested using MTT, cell cycle and Annexin V/PI assays on human MCF-7 and MDA-MB231 breast cancer cells. Mice were orally fed with nordamnacanthal daily for 28 days for oral subchronic toxicity study. Then, the in vivo anti-tumor effect was evaluated on 4T1 murine cancer cells-challenged mice. Changes of tumor size and immune parameters were evaluated on the untreated and nordamnacanthal treated mice.

**Results:**

Nordamnacanthal was found to possess cytotoxic effects on MDA-MB231, MCF-7 and 4T1 cells in vitro. Moreover, based on the cell cycle and Annexin V results, nordamnacanthal managed to induce cell death in both MDA-MB231 and MCF-7 cells. Additionally, no mortality, signs of toxicity and changes of serum liver profile were observed in nordamnacanthal treated mice in the subchronic toxicity study. Furthermore, 50 mg/kg body weight of nordamncanthal successfully delayed the progression of 4T1 tumors in Balb/C mice after 28 days of treatment. Treatment with nordamnacanthal was also able to increase tumor immunity as evidenced by the immunophenotyping of the spleen and YAC-1 cytotoxicity assays.

**Conclusion:**

Nordamnacanthal managed to inhibit the growth and induce cell death in MDA-MB231 and MCF-7 cell lines in vitro and cease the tumor progression of 4T1 cells in vivo*.* Overall, nordamnacanthal holds interesting anti-cancer properties that can be further explored.

## Background

Breast cancer is the leading cause of cancer-related deaths in women today. In Malaysia, the number of cases of women being diagnosed with breast cancer has risen alarmingly, similar to the global trend [1]. Breast cancer can be classified as non-invasive breast cancer and invasive breast cancer. Recent advances in cancer genomics mapping has helped to classify breast cancer based on the expression of cellular receptors, which are estrogen receptor (ER), progesterone receptor, and anti-human epidermal growth factor receptor 2 (HER2) [2]. Various treatments have been used to treat breast cancer including surgery, chemotherapy and radiation [3]. Nevertheless, the number of successful treatments is still limited, mainly due to the severe side effects produced by the treatment [4]. Most of the drugs given to treat breast cancer, or any kind of cancer, may induce unwanted toxic effects. Among different types of breast cancer classified by the expression of ER, PR and ER, triple-negative breast cancer (TNBC) which lacks the expression of all the above cellular receptors is the highly metastatic stage IV breast cancer [2]. The ability of the cells to invade other sites of the body is the main contributor to the number of cancer-associated fatalities [1]. Current research is still attempting to discover better treatment against this advance stage of breast cancer [5]. An ideal treatment would not only inhibit the growth of the cells but also obstruct the metastasis process and enhance the immune system as well. Among the available cell lines, 4T1 cell line has been widely used as a model for in vitro and in vivo study of TNBC [5].

Over the decades, much effort has been directed to using naturally derived molecules as a source of anti-cancer agents [6, 7]. Natural sources have been proven to provide a large database for the search of new drugs [6, 7]. In fact, some of the most remarkable drugs currently used were derived from various natural products including doxorubicin, taxol and curcumin [7]. *Morinda citrifolia* can be found in different parts of the world mainly Borneo, Indonesia, Malaysia and some parts of Australia [8, 9]. This plant is part of the *Rubiaceae* family and can be physically identified as having large, green, shiny leaves [8, 9]. In Malaysia, the fruits of *Morinda citrifolia* are known as *mengkudu* or *noni* [8]. *Mengkudu* is commonly eaten raw or can be used in various local dishes as garnish. Traditionally, the fruits can be turned into juices and be used to treat various illnesses including diabetes and inflammation [10, 11]. In fact, in traditional Chinese medicine, the fruits have been used to treat abdominal pain and menstrual-related diseases [9]. In Hawaii, the roots and barks of *Morinda citrifolia* is traditionally used as dyes [12]. Moreover, besides the leaves and fruits, the roots and barks of this plant are also traditionally used to treat inflammation or infections [12]. There are various bioactive molecules that can be extracted from the stems and roots of the plant but the most notable ones are damnacanthal and nordamnacanthal [13]. Nordamnacanthal is an anthroquinone that can be found in the stems and roots of *Morinda citrifolia* [14]. The bioactivities of nordamnacanthal have been reported but are very preliminary. These reports claim that nordamnacanthal possess anti-viral, anti-microbial and cytotoxic effects [14–16]. The toxicity as well as the effectiveness of nordamnacanthal as an anti-cancer agent in an in vivo setting has not been reported yet. Therefore, this study aims to evaluate the toxicity of nordamncanthal as well as the ability of the compound to inhibit cancer progression in both in vitro and in vivo breast cancer settings.

## Methods

### Isolation of Nordamnacanthal

**Fig. 1:**
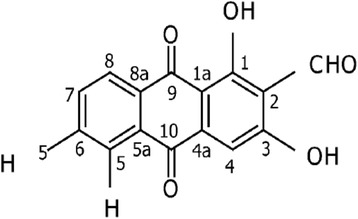
Molecular structure of Nordamnacanthal

*Morinda citrifolia* L. was collected from Kg. Tanjung Keramat, Langkap, Perak, Malaysia. The plant was formally identified by Prof. Dr. Nor Hadiani Ismail (UiTM, Malaysia). Voucher specimen (ATCL 0012) was deposited for future evidence in the herbarium collection. Nordamnacanthal (NDAM) (Fig. 1) was isolated from the root of *M. citrifolia* L. by solvent fractionation. The compound was then purified using high performance liquid chromatography method and characterized as reported in the previous publication [17].

### Cell culture and maintenance

MCF-7, MDA-MB231 and 4T1 cells were obtained from the American Tissue Culture Collection (ATCC, Manassas, USA). Both MCF-7 and 4T1 cells were maintained in RPMI-1640 medium (Sigma-Aldrich, St. Louis, USA) while MDA-MB231 cells were cultured in DMEM medium (Sigma-Aldrich, St. Louis, USA). Both media were supplemented with 10% fetal bovine serum (Cat number: 16,000,044; US origin, Standard Sterile-Filtered; Endotoxin level < 5 EU/mL; Hemoglobin level < 10 mg/dl) (Gibco,Thermo Fisher Scientific, Waltham, USA) and 1% penicillin-streptomycin (Gibco, Thermo Fisher Scientific, Waltham, USA). All of the cells were maintained in a 37 °C humidified CO_2_ incubator equipped with 5% CO_2_.

### In vitro MTT and trypan blue cell viability assays

MCF-7, MDA-MB231 and 4T1 cells were seeded in 96-well plates at the density of 0.8 × 10^4^ cells/well and were left to incubate for 24 h. Seeding of 4T1 cell in 96 well plates were based on the optimization for the cell confluency, where 4T1 cells reached 70% of confluency at 24 h and 95% of confluency at 72 h (results not shown). The following day, various concentrations of NDAM were administered to the cells ranging from 30 μg/mL to 3.75 μg/mL with 2 fold serial dilution for MTT assay and trypan blue cell counting. The cells were incubated for 72 h before assessing the viability of the cells.

After the designated incubation time, 20 μl of MTT solution (5 mg/mL) (Calbiochem, Merck Millipore, Billerica, USA) was added to each of the wells and the plates were further incubated for an additional 4 h. Next, the medium as well as the MTT solution were removed from the wells, and 100 μL of DMSO was added to solubilize the resulting crystals. Lastly, the absorbance of each of the wells was measured using a microplate reader (Biotek Instruments, Winooski, USA) at 570 nm. The percentage of viability was calculated based on the formula below:

Percentage of cell viability (%) = OD Treated/OD Control × 100%.

For trypan blue cell counting, cells were harvested after 72 h of incubation and counted under inverted light microscope (Nikon, Minato, Japan) using hemocytometer (Sigma-Aldrich, St. Louis, USA). The percentage of viability was calculated based on the formula below:

Percentage of cell viability (%) = NDAM treated cell number/untreated cell number × 100%.

### In vitro flow cytometry analyses of cell death (cell cycle and Annexin V)

MCF-7 and MDA-MB231 cells were seeded in 6-well plates at the density of 2.4 × 10^5^ cells/well. The following day, the cells were treated with 10 μg/mL of NDAM for 48 h. After the incubation period, the cells were harvested and subjected to two flow cytometry analysis: Annexin V assay and cell cycle assay. For the Annexin V assay, according to the manufacturer’s protocol (Becton Dickinson, Franklin lakes, USA), the cells were stained with 5 μl of Annexin V-FITC and 5 μl of Propidium Iodide (PI) in 100 μl of 1X Annexin Binding buffer for 15 min. Cell cycle assay was performed using BD cell cycle kit (Becton Dickinson, Franklin lakes, USA) according to the manufacturer’s protocol.In brief, the cells were washed with 1X washing buffer three times, before adding solution 1, solution 2 and solution 3 intermittently. For AnnexinV-FITC/PI assay, all samples were analyzed by FACS Calibur flow cytometer system (Becton Dickinson, Franklin lakes, USA) with BD CellQuest Pro software (Becton Dickinson, Franklin lakes, USA) using four parameters (FSC, SSC, FITC and PE fluorescence). Untreated MCF-7 and MDA-MB231 cells without Annexin V-FITC and PI staining were used as the negative control for gating of auto-fluorescent signal. NDAM (10 μg/mL) treated MCF-7 and MDA-MB231 cells were stained with either Annexin V-FITC or PI and run independently for gating of Annexin V^+^ and PI^+^ cell population. In terms of cell cycle, all samples were analyzed using FACS Calibur flow cytometer system (Becton Dickinson, Franklin lakes, USA) with BD CellQuest Pro software (Becton Dickinson, Franklin lakes, USA) using three parameters (FSC, SSC and PI fluorescence). Untreated MCF7 and MDA-MB231 cells were used for gating of G1, S and G2 cell cycle phases based on the intensity of the red PI fluorescent. For both assays, approximately 10,000 events were collected for each samples based on the optimized gating procedures as described above.

### Animal ethics approval

All studies involving animals were conducted in compliance with the Universiti Putra Malaysia’s ethical guidelines as approved by the Animal Ethics Committee (Universiti Putra Malaysia, Malaysia). The approval number obtained: UPM/IACUC/AUP-R098/2014.

### In vivo subchronic toxicity study

Subchronic toxicity study was performed using 8-weeks old, male BALB/c mice that were obtained from the animal house, Monash University, Malaysia (Subang Jaya, Malaysia). The mice were randomly selected and grouped into 5 mice per group; control group, low dose nordamnacanthal (LD NDAM) group and high dose (HD NDAM) group. All of the mice were kept at 25 ± 2 °C on a regular 12-h dark-light cycle and were fed with tap water and standard diet pellets. The experiment began 1 week after obtaining the mice, allowing the mice to adapt to the laboratory environment. The mice were housed in standard polypropylene cages sized 7 × 10 inches, with 4-5 mice per cage. The treatment of 10 mg/kg/day of NDAM for the low dose nordamncanthal (LD NDAM) group and 50 mg/kg/day for the high dose (HD NDAM) groupwere administered orally for 28 days. The treatments were administered consistently at 11 am every day. Toxicity signs such as shedding of fur, loss of appetite and erratic behavior were observed daily if there are any. At the end of the 28 days of experimental period, all mice were anesthetized with isoflurane (Sigma-Aldrich, St. Louis, USA), and euthanized by cervical dislocation.

### Serum biochemical analysis

Blood was obtained from the sacrificed mice by cardiac puncture and the serum was obtained by centrifugation. Next, the serum was subjected to biochemical analysis. The level of aspartate aminotransferase (AST), alanine aminotransferase (ALT), alkaline phosphatase (ALP), creatinine, and albumin, in mice serum were determined by standard assay kits (Roche Diagnostic GmbH, Indianapolis, USA) and analyzed using 902 Hitachi automatic analyser (Hitachi LTD, Chiyoda, Japan).

### In vivo study of the antitumor effect of NDAM using 4T1-bearing BALB/C mice

For the in vivo study of the antitumor effect of NDAM, 8-weeks old, female BALB/C mice were obtained from animal house of Monash University Malaysia (Subang Jaya, Malaysia). The mice were acclimatized to the laboratory environment for 1 week before commencing the experiment. Around 1 × 10^5^ of 4T1 cells were inoculated in the mice subcutaneously and then, the mice were randomly divided into two groups; control and NDAM groups with each group bearing 6 mice. Tumours were measured using a caliper and the tumour volumes were calculated using the formula V = 1/2 (width^2^× length).For the treatment, 50 mg/kg of NDAM was administered orally to the mice from day 5 when the tumor volume reach around 0.5mm^3^ until day 28. After the designated treatment time, all mice were anesthetized with isoflurane (Sigma-Aldrich, St. Louis, USA), and euthanized by cervical dislocation The organs were harvested for further analyses.

### Immunophenotyping analysis of CD3, CD4 and CD8-stained splenocytes

Spleens harvested from mice from each groups were mechanically disrupt by meshing the organs using 70 μmeter cell strainer. The cells were then washed with ice-cold PBS twice before being subjected to lysis buffer for 15 min to remove red blood cells. Subsequently, the cells were washed with PBS again before being stained with anti-CD3-FITC [UCHT1] with anti-CD4-PE [74-12-4] or anti-CD3-FITC [UCHT1] with anti-CD8-PerCP [MEM-31] antibodies (Abcam, Cambridge, UK; Catalog No: ab34275, ab25408, ab65949).The cells were incubated for 2 h at 4 °C before being fixed in 4% paraformaldehyde. On the next day, the stained cells were washed and run by FACS Calibur flow cytometer system (Becton Dickinson, Franklin lakes, USA) with BD CellQuest Pro software (Becton Dickinson, Franklin lakes, USA) using five parameters (FSC, SSC, FITC, PE and PerCP fluorescence). Splenocytes from untreated 4T1-mice stained with isotype-control IgG (Abcam, Cambridge, UK; Catalog no: ab37355) was used for the gating of auto-fluorescent signal. Splenocytes from NDAM treated 4T1-mice was stained with single antibody of either anti-CD3-FITC, anti-CD4-PE or anti-CD8-PerCP for establishment of gating of the double staining. Above compensation was used to run the splenocytes from untreated 4T1-mice and NDAM treated 4T1-mice and approximately 10,000 events were collected for each samples based on the optimized gating procedures as described above.

### Cytokine analysis of IL-2, IL-4 and IFN-γ

Cytokine analysis of IL-2, IL-4 and IFN-γ in the serum of the untreated and NDAM-treated 4T1-mice was performed using the ELISA IL-2, IL-4 and IFN-γ MAX™ kit (Biolegend, San Diego, USA). Samples from normal mice treated with nordamnacanthal (50 mg/kg body weight) was collected from the female mice via tail vein sampling at day 28 of feeding with 50 mg/kg body weight of nordamnacanthal from subchronic toxicity test. Briefly, antibodies for IL-2, IL-4 and IFN-γ were fixed in the wells of 96-well plates overnight. The following day, after series of washing and blocking, samples were incubated in the plates for 2 h. Subsequently, the wells were then stained and measured colorimetrically using a microplate reader (Biotek Instruments, Winooski, USA). The value of absorbance of each sample was calculated against the respective control.

### Ex vivo splenocytes and YAC-1 co-culture analysis

Spleens harvested from mice from each groups were mechanically disrupt by meshing the organs using 70 μmeter cell strainer (Becton Dickinson, Franklin lakes, USA). The cells were then washed with ice-cold PBS (Sigma-Aldrich, St. Louis, USA) twice before being subjected to lysis buffer for 15 min to remove red blood cells. The splenocytes were counted and were seeded together with YAC-1 cells at a ratio of 1 to 5 and 1 to 10. After 24 h, the cytotoxicity was evaluated using the CytoTox 96 nonradioactive cytotoxicity assay kit (Promega, Madison,USA) according to the manufacturer’s protocol.

### Statistical analysis

In vitro experiments were carried out with 3 independent experiments and each of the experiment consisted of at least 3 biological replicates. In vivo experiment was assayed on all 6 mice and each with 3 technical replicates. All results are expressed as Mean ± Standard Deviation (S.D.). Significant levels (*p* < 0.05) were evaluated using ANOVA test (one way) followed by post hoc Duncan test.

## Results

### NDAM inhibited the proliferation and induced apoptosis in MCF-7, MDA-MB231 and 4T1 cells in vitro

**Fig. 2:**
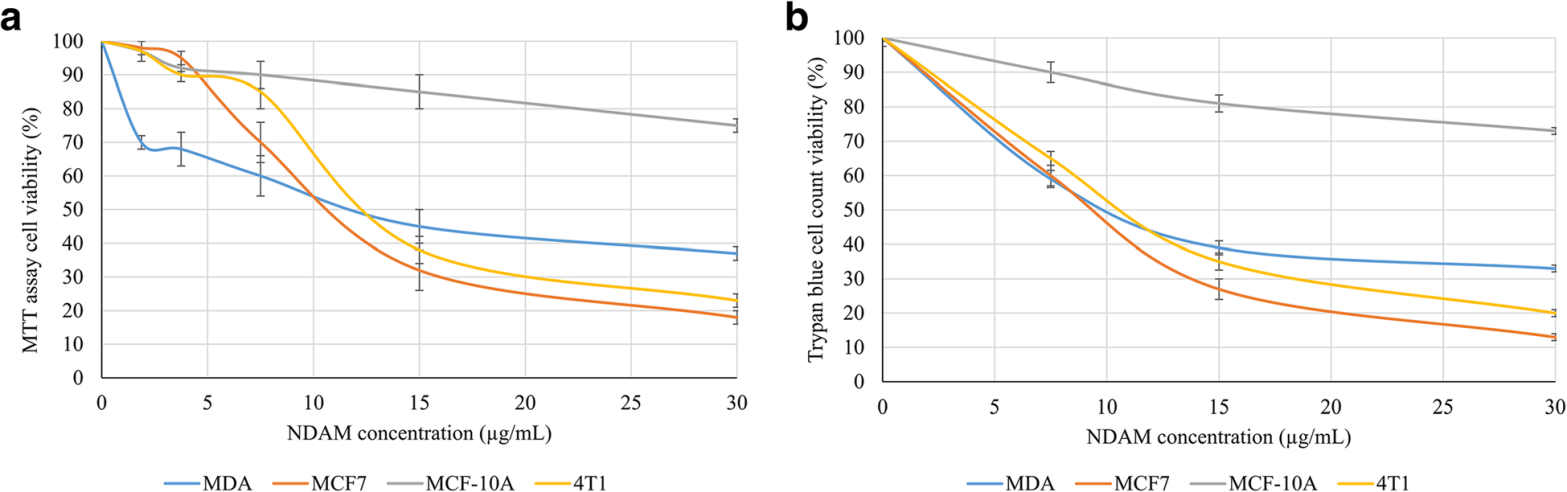
Percentage of viable cells against different concentrations of NDAM in MCF-7, MDA-MB231, 4T1 and MCF-10A cells after 72 h of treatment quantified by (**a**) MTT assay and (**b**) Trypan blue cell counting

**Fig. 3:**
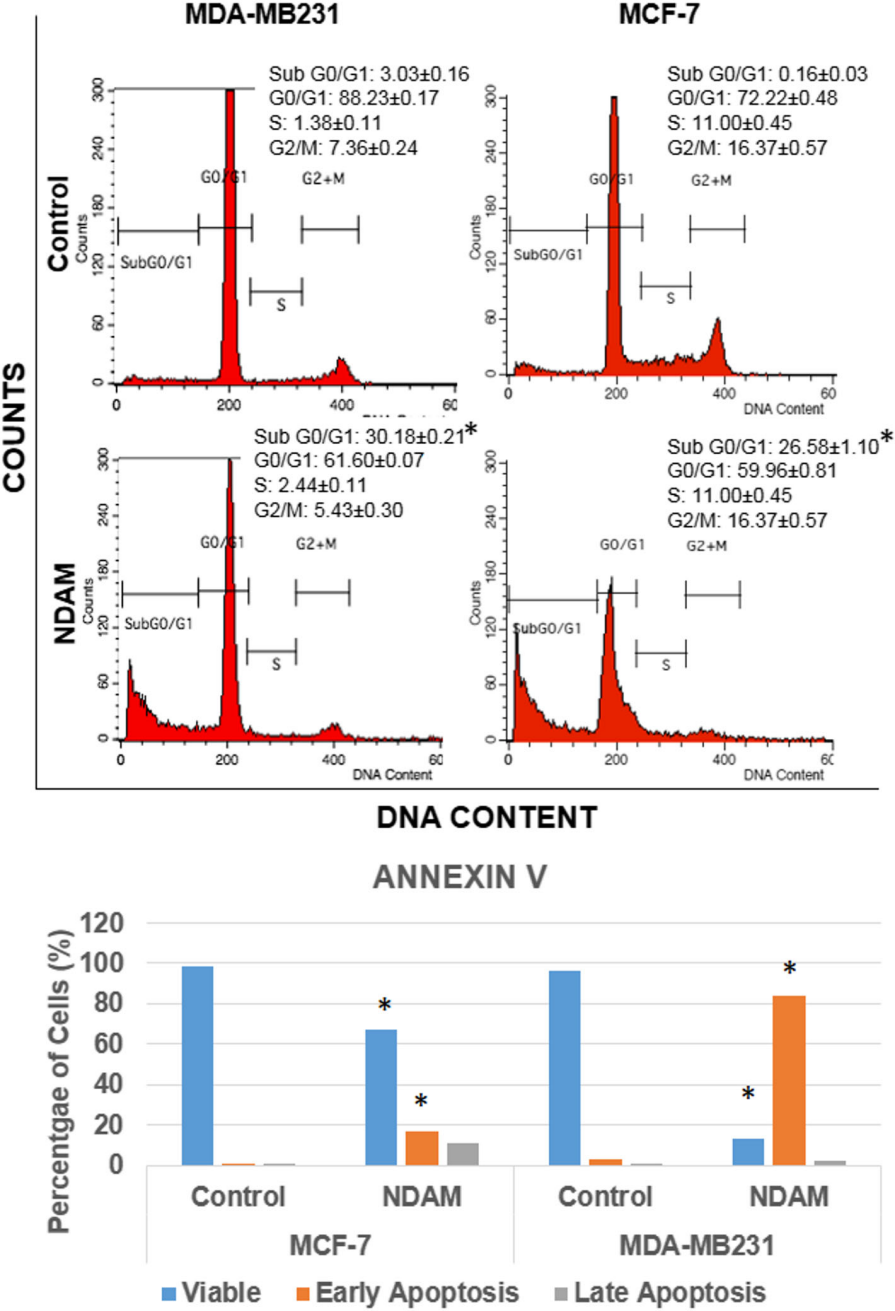
Cell cycle analysis and Annexin V analysis in MCF-7 and MDA-MB231 cells after treatment with 10 μg/mL of NDAM for 48 h. Values represent the mean with standard deviation. *Significance set at *p* < 0.05

**Fig. 4:**
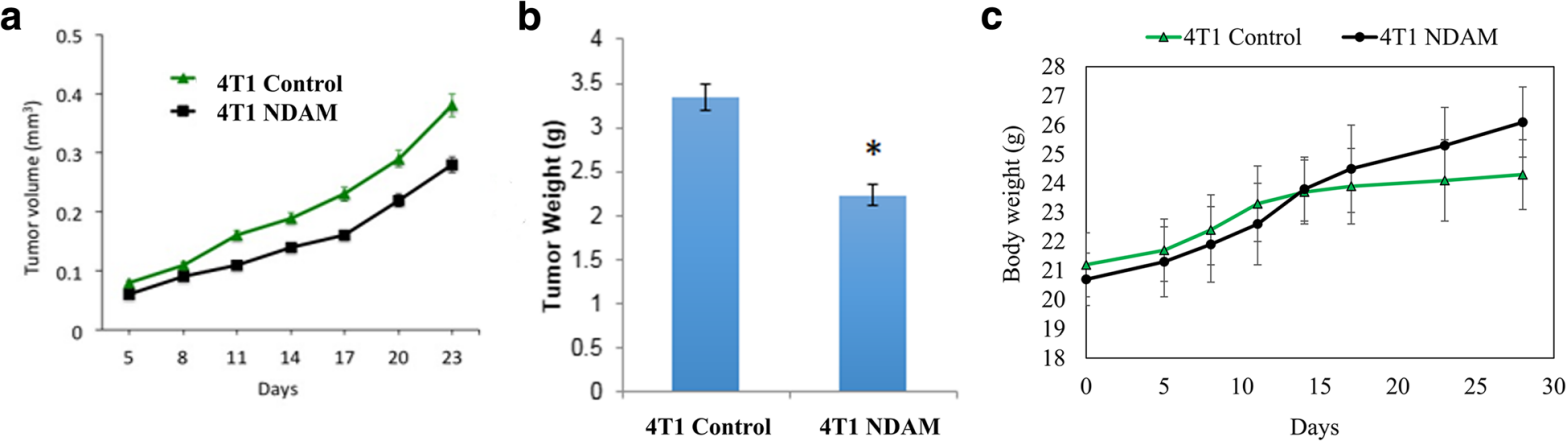
**a** Tumor volume; **b** tumor weight and (**c**) body weight of control and NDAM-treated 4T1-mice after 28 days of treatment. Values represent the mean with standard deviation. *Significance set at *p* < 0.05

The MTT assay was carried out as a preliminary testing of the cytotoxic effects of NDAM on various cancer cell lines. Based on both MTT assay (Fig. 2a) and Trypan blue cell counting (Fig. 2b), NDAM managed to reduce the viability of MCF-7,MDA-MB231 and 4T1 cells in a dose-dependent manner as higher concentration of NDAM reduced higher degree of the viability for all the tested breast cancer cells. In the MTT assay, the IC_50_ value of NDAM on MDA-MB231 and 4T1 cells was almost similar, around 12.5 ± 4.2 μg/mL. Meanwhile, the IC_50_ value for MCF-7 cells was around 11.0 ± 4.7 μg/mL (Fig. 2a). When tested with Trypan blue cell counting, IC_50_ values of NDAM on MCF-7, MDA-MB231 and 4T1 were slightly lower than MTT assay with 8.0 ± 1.2 μg/mL, 10.0 ± 0.8 μg/mL, 11.0 ± 1.0 μg/mL, respectively. No IC_50_ value was obtained on normal breast cells MCF10A treated with NDAM in both MTT assay and trypan blue cell counting. Furthermore, additional assays were carried out to elucidate the mechanism of cell death triggered by NDAM. Based on the Annexin V assay in Fig. 4, NDAM increased the population of early apoptosis and late apoptosis cells in both MCF-7 and MDA-MB231 as compared to the control after 48 h of treatment. Additionally, the profile of cell cycle analysis also exhibited the same pattern. The number of cell population at the Sub G0/G1 phase increased significantly in the NDAM-treated cells for both MCF-7 and MDA-MB231 cells as shown in Fig. 3.

### NDAM did not induce any toxic effects in the in vivo subchronic toxicity study

Based on Table 1, NDAM did not cause any deaths in the mice after 28 days of treatment with two different doses of treatment. Moreover, there were no apparent toxic signs observed and no significant changes to the body weight of the mice tested. Biochemical analysis of important organ markers were also tested in the serum of the mice tested. After 28 days, there were no significant changes to the values of ALT, AST and ALP levels comparing to the untreated control.

**Table 1:** Evaluation of the sub-chronic toxicity of NDAM in BALB/C mice

	Mortality	Body weight (g) Day 0	Body weight (g) Day 28	Body weight (g) Day 60	Toxic Signs	ALT (U/L)	AST (U/L)	ALP (U/L)
Control	None	20.30 ± 1.10	23.10 ± 1.70	25.60 ± 1.80	None	75.6 ± 34.0	301.5 ± 53.0	101.7 ± 13.0
LDNDAM (10 mg/kg)	None	19.86 ± 1.30	23.85 ± 1.90	25.80 ± 1.70	None	69.4 ± 39.0	316.0 ± 37.0	125.4 ± 20.4
HDNDAM (50 mg/kg)	None	20.80 ± 1.40	22.50 ± 2.10	24.90 ± 2.30	None	64.5 ± 32.0	246.0 ± 55.0	113.7 ± 19.0

Parameters evaluated for sub-chronic toxicity test including mortality rates, body weight, toxic signs and biochemical analysis of ALT, AST and ALP

No statistically significant was observed compared to control (*p* < 0.05)

### NDAM reduced the 4T1 tumor size and weight

After 28 days of treatment with 50 mg/kg of NDAM in female BALB/C mice, the mice were sacrificed and the tumors and organs were harvested. In the duration of the treatment, no mortalities were observed, all of the mice survived until the end of the experiment. In addition, no significant body weight changes were observed between control and NDAM treated 4T1-mice within 28 days of study period (Fig. 4c). Tumors were harvested from both the control and NDAM-treated 4T1 group were weighed and measured individually. As shown in Fig. 4a the average volume of the tumors from the NDAM-treated 4T1-mice were smaller in relative to the control group throughout the experiment. The same pattern can also be observed in the weight of the harvested tumors as displayed in Fig. 4b.

### NDAM regulated several immune markers in tumor-bearing mice

**Fig. 5:**
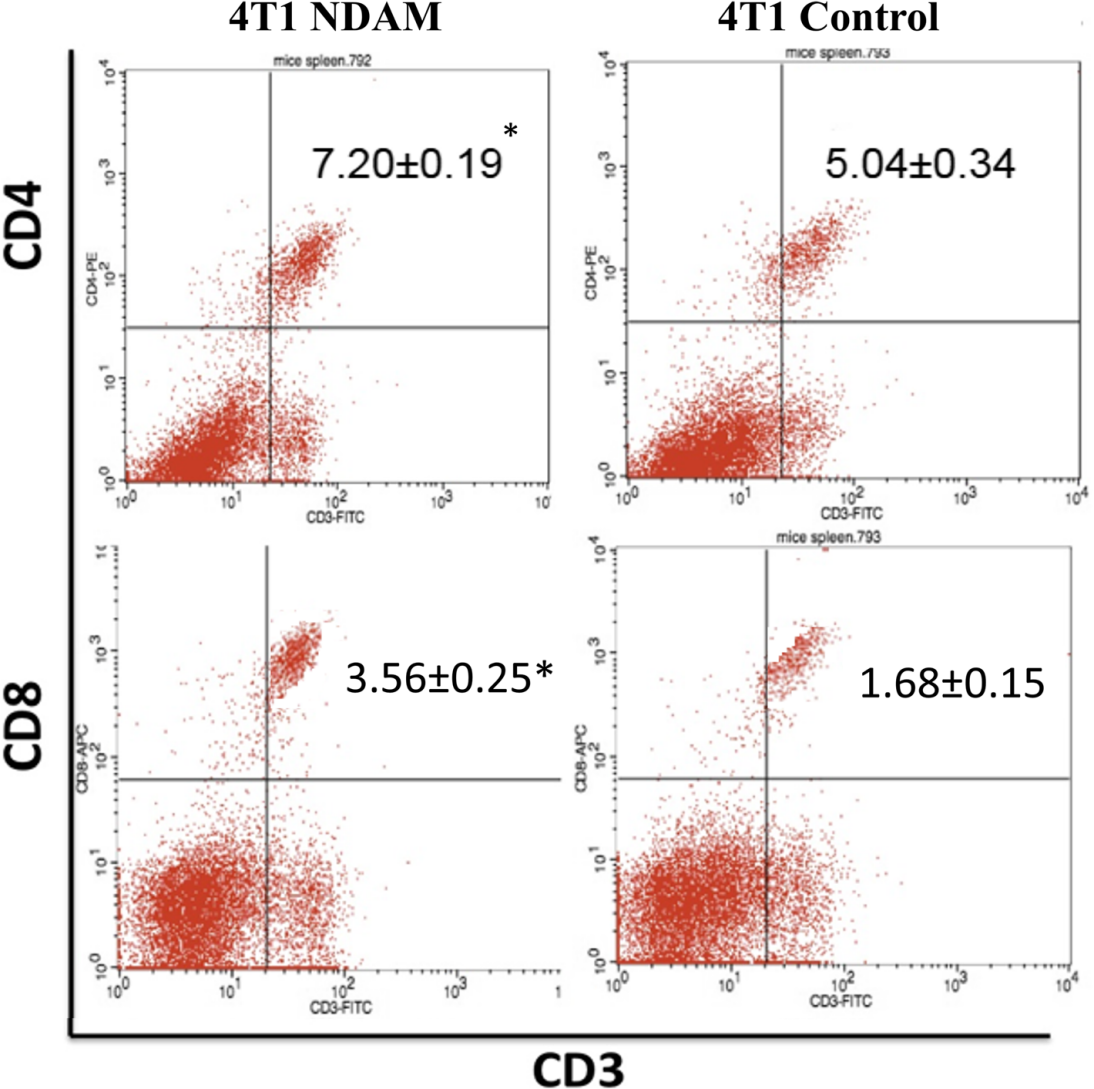
Percentage of the immunophenotyping results of the spleens harvested from the control and NDAM-treated 4T1-mice. Values represent the mean with standard deviation. *Significance set at *p* < 0.05

**Fig. 6:**
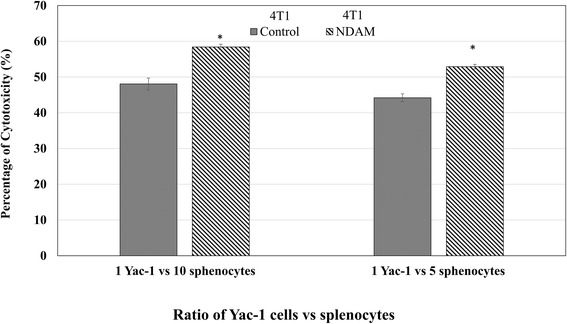
Cytotoxicity values for the co-culture of splenocytes and YAC-1 cells.Values represent the mean with standard deviation. *Significance set at *p* < 0.05

**Fig. 7:**
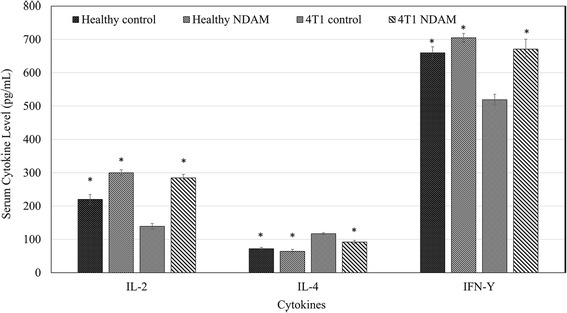
Concentration of cytokines IL-2 and IFN-γ from the serum of the mice from both the healthy control, healthy NDAM, untreated 4T1 and 50 mg/kg NDAM-treated 4T1 mice after 28 days of treatment. Values represent the mean with standard deviation. *Significance set at *p* < 0.05

The level of T cells was measured in the splenocytes of the control and NDAM-treated 4T1-mice via immunophenotyping. As in Fig. 5, the level of CD4/CD3 cells was increased in NDAM-treated 4 T1-mice as compared to the control mice. Similarly, the level of CD8/CD3 cells was also elevated in the NDAM-treated 4T1-mice than the untreated mice. Additionally, there was no significant difference in the level of NK1.1/CD3 cells between the NDAM-treated 4T1-mice and the control mice. Nevertheless, based on the cytotoxicity results of splenocytes against YAC-1 cells, NDAM-treated splenocytes had a higher percentage of toxicity using both 1:10 and 1:5 ratios as in Fig. 6. Moreover, in the serum of the NDAM-treated 4T1-mice, the concentration of IL-2 and IFN-γ were significantly higher than the untreated mice (Fig. 7). On the other hand, level of serum IL-4 was lower in NDAM-treated 4T1-mice. The IL-2, IL-4 and IFN-γ of the NDAM-treated 4T1-mice are similar with the healthy control and healthy NDAM mice without significant changes (Fig. 7).

## Discussion

*Morinda citrifolia* is a well-known plant that possesses various promising bioactivities. A notable anthraquinone, damncanthal, which can be extracted from this plant has been known to be involved in several anti-cancer mechanisms. A lesser known anthraquinone is the NDAM, which can also be extracted from the stems of *Morinda citrifolia*. Anthraquinones are an interesting set of molecules that can either be synthetically produced or found in nature naturally. This set of molecules is known to be used in several applications namely; anti-malarial drugs, anti-neoplastic drugs and colorants in food, textile and cosmetics [18, 19]. Anthraquinones have immensely contributed to the development of effective anti-cancer drugs [20]. NDAM has been reported to be cytotoxic to several cancer cell lines based on the MTT assay [14–16]. Herein, NDAM was shown to be cytotoxic to all three breast cancer cell lines, MCF-7, MDA-MB231 and 4T1 cells. It has been reported that NDAM also inhibited the proliferation of several other cancer cell lines such as HL-60, CEM-SS, WEHI-3B and K562 [14, 15]. Nevertheless, the IC_50_ values of NDAM in MCF-7 and MDA-MB231 were much lower than the other reported cell lines. The effects on MCF-7 and MDA-MB231 were similar despite the differences between the two cell lines. NDAM managed to induce apoptosis in both cell lines as evidenced by the Annexin V and cell cycle analysis. The externalization of phosphatidylserine is a vital parameter in cells undergoing apoptosis and has been used to support the induction of apoptosis by using Annexin V-based assays [21]. Furthermore, the deregulation of the cell cycle process is also an imperative step in the execution of apoptosis [22, 23]. Inhibition of cell proliferation could tilt the balance between cell survival and cell death and eventually trigger tumor regression [22]. Damnacanthal, a similar molecule to NDAM also exhibited striking anti-tumorigenesis effects in vitro by inducing apoptosis in several different cancer cell lines. Though damnacanthal has a much lower IC_50_ value in MCF-7 and MDA-MB231 than NDAM, damnacanthal has been reported to be less selective in non-transformed mammary cell line, MCF-10A [24].

Though anthraquinones have a wide range of applications, there is still concern regarding the safety of anthraquinones especially for human consumption [19]. Therefore, it is important to measure the toxicity of NDAM before applying it for other purposes. Ideally, a safe sample would not induce any mortality, toxic signs or severe body weight changes to the subjects tested. Besides that, the levels of ALT, ALP and AST are also important indicators for hepatic injury. Leakage of these proteins in the blood could indicate damage to hepatocytes such as loss of membrane integrity and mitochondrial damage and could serve as a sign of toxicity [25]. NDAM was shown to not be toxic on the mice tested after 28 days based on the physical appearance of the mice and the levels of AST, ALT and ALP.

The efficacy of a treatment in vitro cannot be directly translated into a more complex setting. As NDAM did not induce any sub-chronic toxicity, in vivo antitumor study using 4T1 cells was further conducted. Inoculation of 4T1 cells in mice is a well-established method for in vivo models of breast cancer. The size and weight of the tumors are basic representation of the effectiveness of the treatment administered. Logically, a specific treatment should reduce the size and weight of the harvested tumors as compared to the control. Additionally, the effect between immune markers and cancer progression is greatly linked. A healthy and active immune system could enhance the anti-cancer treatment and thus reducing the progression of cancer [26]. NDAM managed to enhance the percentage of CD8/CD3 and NK cells as evidenced by the immunophenotyping and cytotoxicity against YAC cells. Both cytotoxic T cells and NK cells defend the host by lysing tumor cells upon recognition [26–28]. Cytotoxic T cells is known to induce cell death via granzyme B-mediated apoptosis [29]. Additionally, CD4/CD3 population and Type 1 T helper (Th1) associated cytokines including IL-2 and IFN- γ were also increased upon NDAM treatment. On the other hand, level of IL-4, a type 2 T helper (Th2) associated cytokine was found reduced in the NDAM-treated 4T1 mice. These results indicated that NDAM treatment increased Th1/Th2 ratio. Th1 cells function by maintaining the activity of T cytotoxic cells and memory of the immune system [30]. Moreover, Th1 cells are able to recruit several other important immune players such as mast cells and macrophages [30]. Activation of Th1 and Th1 associated cytokines including IFN-γ and IL-2contribute to the host’s defense mechanism against cancer [30–32].

## Conclusion

Despite the progress in cancer research, the search for viable anti-cancer drug is still on the rise. NDAM, an anthroquinone that can be found in the stems of *Morinda citrifolia* is a promising compound that possessed remarkable anti-cancer properties. Based on this pilot in vivo study, NDAM was not toxic in the animals tested. Moreover, nordamnacanthal managed to reduce the viability of two breast cancer cells, MCF-7 and MDA-MB231 in vitro*.* NDAM successfully decreased the size of the 4T1 tumors in vivo and increased the population of T helper, cytotoxic T, and NK cells. Further studies using larger sample size and different models of antitumor studies can further support the potential of NDAM for treatment of breast cancer.

## Abbreviations

ALP: Alkaline Phosphatase
ALT: Alanine Aminotransferase
AST: Aspartate Aminotransferase
ER: Estrogen Receptor
HD: High dose
HER2: Anti-human Epidermal Growth Factor Receptor 2
IL: Interleukin
LD: Low dose
MTT: 3-(4,5-dimethylthiazol-2-yl)-2,5-diphenyltetrazolium bromide
NDAM: Nordamnacanthal
PI: Propidium Iodide
SD: Standard Deviation
Th2: T helper
TNBC: Triple-negative breast cancer
